# Influence of Climate on Radial Growth of Black Pine on the Mountain Regions of Southwestern Turkey

**DOI:** 10.3390/plants8080276

**Published:** 2019-08-09

**Authors:** Mehmet Doğan, Nesibe Köse

**Affiliations:** 1 Ege University, Faculty of Letters, Department of Geography, 35100 Bornova-Izmir, Turkey; 2 Istanbul University-Cerrahpasa, Faculty of Forestry, Forest Botany Department, 34473 Bahçeköy-Istanbul, Turkey

**Keywords:** black pine, tree-ring, radial growth, dendroecology, response function, Southwestern Turkey

## Abstract

In this study, we identified the most important climate factors affecting the radial growth of black pine at different elevations of the mountain regions of Southwestern Turkey (Sandıras Mountain, Muğla/Turkey). We used four black pine tree-ring chronologies, which represent upper and lower distribution limits of black pine forest on the South and North slopes of Sandıras Mountain. The relationships between tree-ring width and climate were identified using response function analysis. We performed hierarchical cluster analysis to classify the response functions into meaningful groups. Black pine trees in the mountain regions of Southwestern Turkey responded positively to a warmer temperature and high precipitation at the beginning of the growing season. As high summer temperatures exacerbated drought, radial growth was affected negatively. Hierarchical cluster analysis made clear that elevation differences, rather than aspect, was the main factor responsible for the formation of the clusters. Due to the mountainous terrain of the study area, the changing climatic conditions (air temperature and precipitation) affected the tree-ring widths differently depending on elevation.

## 1. Introduction

Tree-rings have been used to determine the effect of environmental factors on tree growth because year-by-year changes in environmental factors cause variations in tree-ring widths. Of environmental factors, climate (especially air temperature and precipitation) is the most important factor affecting tree-ring growth [1,2]. Dendroclimatology has been widely used to understand the effect of climate on radial growth of conifer species such as *Pinus sylvestris* [3,4,5] *Pinus nigra* [6,7,8,9,10], *Pinus strobus* [11], fir [12,13,14], spruce [11,12,14,15], larch [14], and juniper [16].

The spatial distribution of air temperature and precipitation are geographically highly variable due to the continentality, distance to the sea, altitude and orographic/topographic characteristics [17,18]. For this reason, depending on physical geography properties, especially elevation and aspect in mountainous areas, air temperature and precipitation change within a very short distance, which causes the annual ring growth of trees distributed at different elevations and slopes to be different. Further, due to the temperature and precipitation variance based on elevation and slopes, the difference in the length of the vegetative period in the lower and upper limits of the forest causes the annual ring width of the tree to be different [2,19,20]. Moreover, the response of trees distributed at different elevations and on different slopes to temperature and precipitation variation may differ from each other. For example, Mazza et al. [13] analyzed the climate–growth relationships of silver fir along an altitudinal gradient in Central Italy. Their results emphasized different growth responses along the gradient which represent the positive influence of previous late spring-summer precipitation and negative influence of previous and current year summer temperature in optimal conditions while these climate influences disappear at the highest site. Fan et al. [12] analyzed fir and spruce growth response to climate at a high elevation gradient from 3200 to 4000 in southwestern China. Their results showed that winter temperatures were the most important limiting factor for the radial growth of trees at middle and high elevation, while spring moisture availability was essential for the trees at lower sites.

Black pine (*Pinus nigra* JF Arnold) is a Mediterranean pine species with a broad distribution area, from the Iberian Peninsula in the west to Turkey in the east, and its northern limit in Austria [6]. In Turkey, it grows widely across the west of the country, but its distribution is restricted to coastal and mountain habitats [21,22]. Several dendroclimatological and dendroecological studies [6,7,23,24,25,26,27,28,29,30] in the Mediterranean region have focused on black pine, which is a drought-sensitive species. Tree-ring studies in the western Mediterranean (in Spain and France), have shown that a cool, wet autumn and spring and/or mild winter enhance radial growth. In general, total ring growth has been found positively correlated with previous autumn, current May–August precipitation and winter temperature, and negatively correlated with previous October and May–August temperature [9,10,31]. In the eastern Mediterranean (especially in Turkey), old black pine forests have been widely used to reconstruct past climate and streamflow [7,8,23,24,25,26,27,28,29]. All these studies showed that the most important limiting factor on the radial growth of black pine is spring/summer precipitation. In general, total ring growth has been found positively correlated with May–June precipitation. In addition, it has been found that black pine responds positively to a warmer temperature at the beginning of growing season [7,8,23,25,26,28,32,33].

Köse et al. [8] determined and classified limiting factors for the radial growth of black pine using a large tree-ring network which was built mostly for dendroclimatological reconstruction. This research has provided regional scale information about the black pine trees, which are mostly grown at the upper elevations. The old and sensitive trees usually found at the upper elevations and steep and southern slopes, where human effect is relatively less. On the other hand, the studies relating the climate response of the black pine, growing at different elevations and slopes, are quite limited in Turkey [34].

The aims of the present study are: (1) to identify the most important climate factors affecting the radial growth of black pine (*Pinus nigra* JF Arnold subsp. *pallasiana*) on Sandıras Mountain, (2) to determine the relationships between climate and tree-ring widths of trees growing at different elevations and slopes, and (3) to classify trees based on their responses to climate.

## 2. Results

**Figure 1:**
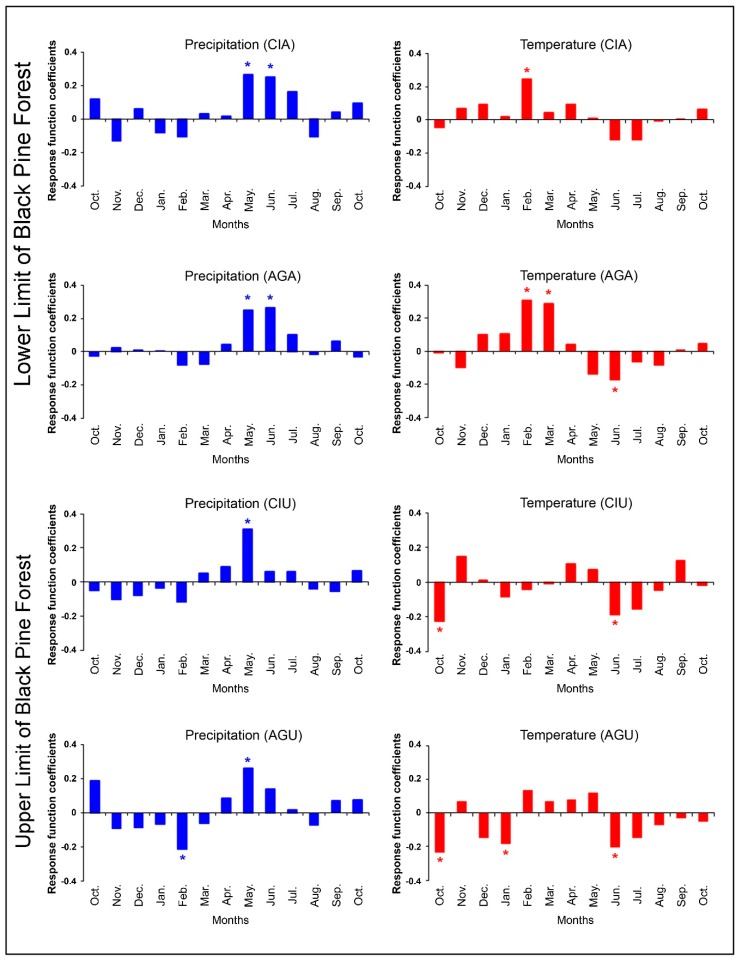
Mean response function coefficients of four site chronologies. Asterisks (*) represent significant correlation coefficient (95% level) with the related month.

Response function coefficients, between the chronologies and climate variables (monthly mean temperature and monthly total precipitation) were given in Figure 1. The effect of precipitation on CIA chronology, obtained from the lower limit of black pine forest on the north slope of the mountain, was positive from May to July and statistically significant in May and June. Higher temperatures in February had a positive and significant effect on radial growth in this site. Response function coefficients related to precipitation were significantly positive only in May for CIU chronology, obtained from the upper limit of black pine forest at north slope of the mountain. The effect of precipitation was very weak for all the other months. On the other hand, the effect of temperature at this site was negative in the previous October and current June. The effect of precipitation on AGA chronology, obtained from the lower limit of black pine forest at south slope of the mountain, was positive and significant in May and June, while the effect of mean temperature was significantly positive in February and March, and significantly negative in June. The effect of precipitation on AGU chronology, obtained from the upper limit of black pine forest at south slope of the mountain, was positive in May. In contrast with the other sites, higher February precipitation had a negative effect on radial growth of black pine in this site. On the other hand, the temperature showed a negative effect in October of the previous year and in January and June of the current year.

**Figure 2:**
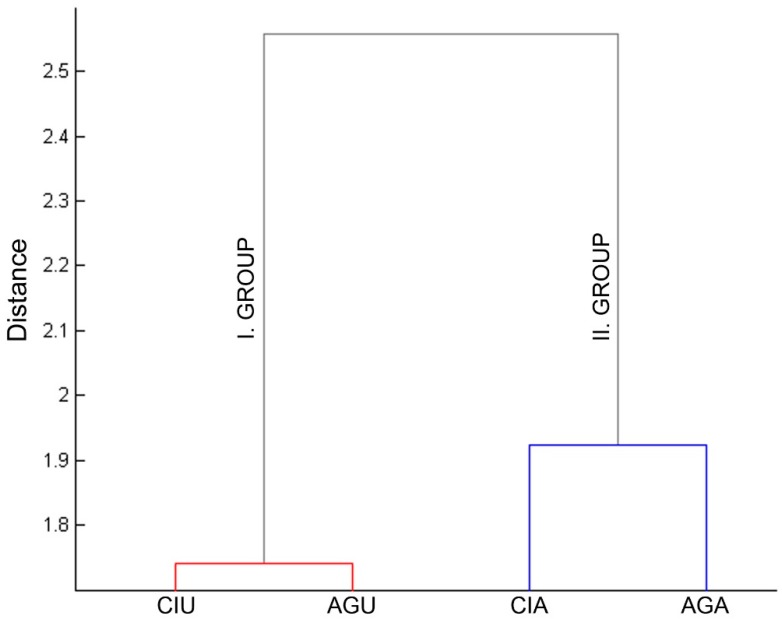
Dendrogram presenting the results from the hierarchical cluster analysis on the response function coefficients of the four site chronologies.

The dendrogram in Figure 2 illustrates that group 1 was composed of the chronologies located in the upper forest limits of the mountain (CIU and AGU), while the site chronologies from lower forest limits (CIA and AGA) were combined into group 2. It is clear that elevation differences rather than slope are the main factor responsible for the formation of the clusters.

## 3. Discussion

It is well known that May–June precipitation is the most important limiting factor on the radial growth of black pine in Turkey [8,24,26,27,35,36,37]. We found similar results for the black pine forest on Sandıras Mountain. Higher precipitation during the period of May–June favoured the production of larger tree-ring at the lower limits of black pine forests (Group 2). On the other hand, the positive effect of precipitation was shown only in May for the upper limits of black pine forests (Group 1). Drought occurrence is a limiting factor for the growth of trees near the lower elevational forest limits [2]. Therefore, we found more distinctive drought effect on the lower limits of black pine forest than the upper limits. Moreover, the lower elevation chronologies in group 2 were more sensitive to climate (mean sensitivity values are 0.27 and 0.22 for CIA and AGA, respectively) than the higher elevation chronologies in group 1 (mean sensitivity values are 0.14 and 0.16 for CIU and AGU, respectively (Table 1).

**Figure 3:**
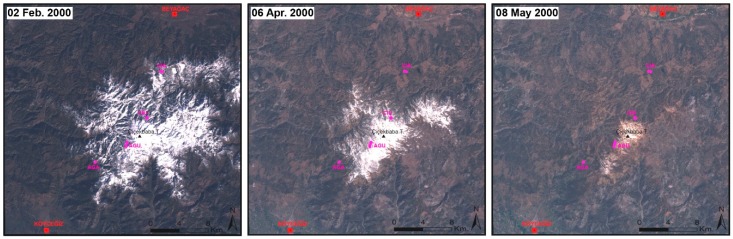
Areas covered with snow at different dates on Sandıras Mountain. Landsat TM imageries (321 band combination) [43]. Magenta areas correspond with the sampling sites. The red points at the upper and lower sides of the images indicate the settlement (city) centers (also see Figure 8).

The only significant negative coefficient related with precipitation was obtained in February for AGU chronology located upper limit on the south slope of the mountain. Köse [15] and Thomsen [38] found a similar response to winter precipitation for Uludağ fir in Kastamonu and for Scots pine in northwestern Siberian Plain, respectively. High snow cover may delay the beginning of the vegetative period at high elevations due to decreasing soil temperature and frost drought, which occur in winter due to low temperatures, and reduce or interrupt water transport [38,39]. However, this negative effect was not significant on the upper limit of black pine forest on the north slope (CIU), even though both sites were located at almost the same elevations. This can be explained by the fact that in our study system, the southern slope of the mountain receives more precipitation (mostly snow in winter) than the northern slope because Sandıras Mountain keeps a large amount of the precipitation, which is brought by the frontal systems over the Mediterranean Sea (from the south) [40,41,42,43]. For a better understanding of response differences between both slopes, we obtained Lansad Satellite Image (NASA) from February to May for the year 2000 (Figure 3). These images show that the south slope receives more snow, and snowpack stays on the ground for longer than on north slope, at the upper limit of black pine.

A high summer temperature and low precipitation increased the drought effect in all sites (Figure 1), having a negative effect on secondary growth. Köse et al. [8] found similar results for black pine trees located in central Anatolia, western Turkey, and the Mediterranean region. The effect of temperature was positive at the beginning of the vegetative period for the lower forest limits (group 2), in February for CIA and in February and March for AGA chronologies.

The effect of temperature was negative in previous October for the upper limit of the black pine forest (AGU and CIU). Akkemik [20] stated that the high temperatures experienced in the early autumn increase the annual ring growth, cause stored nutrients to be consumed and give rise to narrower tree ring the following year. Accordingly, on the upper limit of the black pine forest on Sandıras Mountain, the temperatures above average in October may have led to the continuation of the tree-ring growth, resulting in the investment of the stored nutrients, and as a consequence, tree-ring development in the following year may be limited.

**Figure 4:**
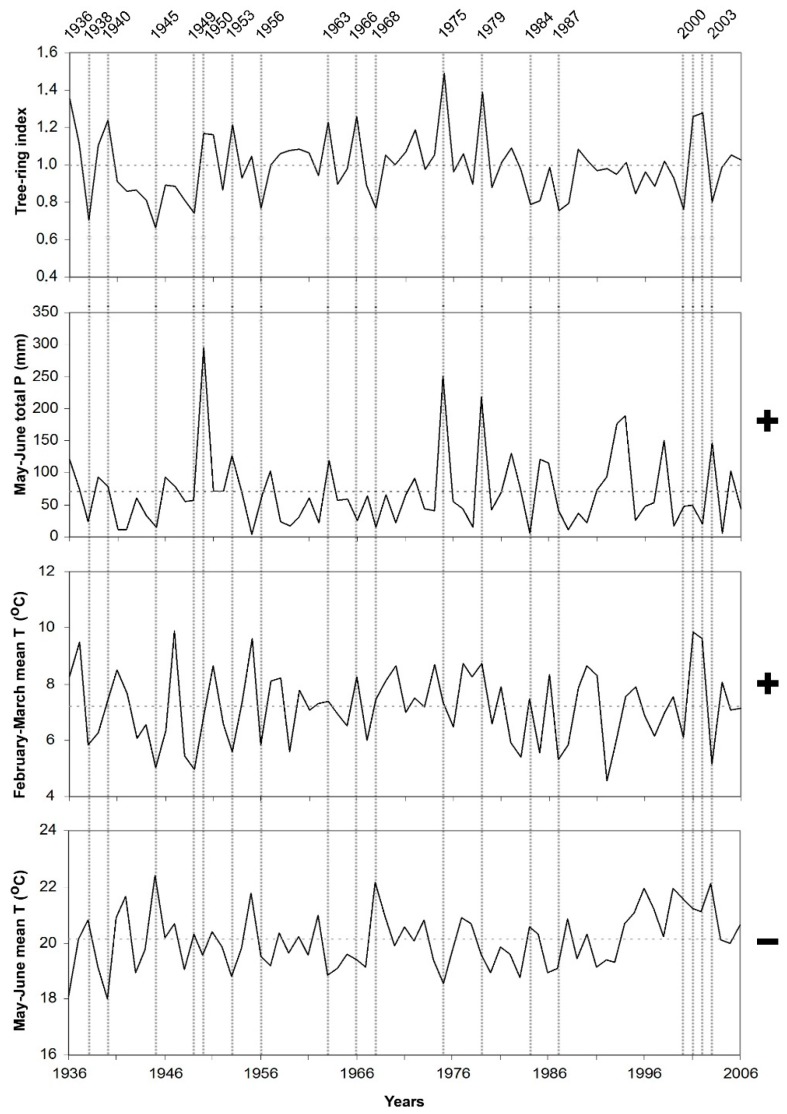
Residual chronology of lower limit of black pine on the south slope (AGA site) and limiting climate factors, which are May–June total precipitation, February–March and May–June mean temperature in the period of 1936–2006. Vertical dashed lines represent pointer years, which have very narrow and large ring formation years. Horizontal dashed lines represent the average value. “+” and “−” represent the positive and the negative correlation between climate factors and tree-ring width, respectively.

**Figure 5:**
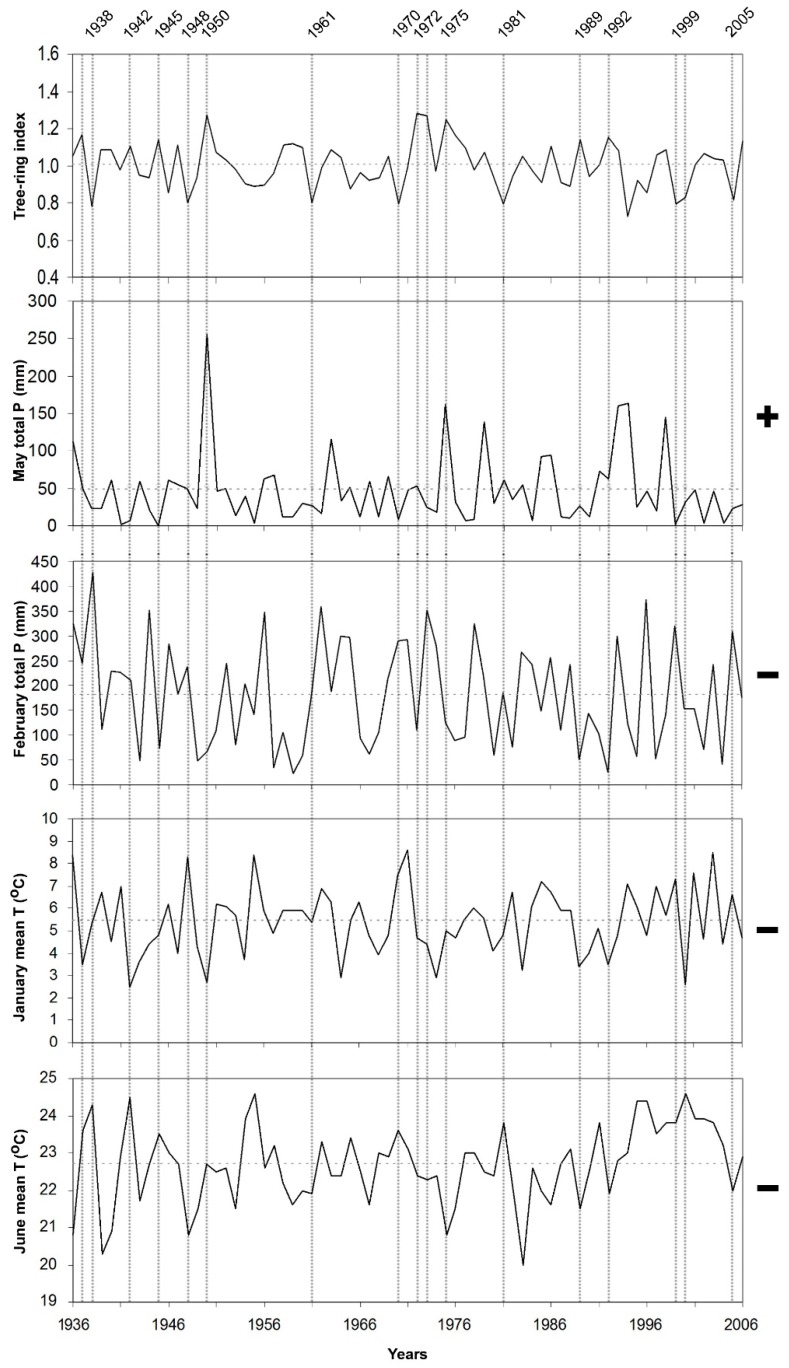
Residual chronology of upper limit of black pine on the south slope (AGU site), and limiting climate factors, which are May total precipitation, February total precipitation, January mean temperature and June mean temperature in the period of 1936–2006. Vertical dashed lines represent pointer years, which have very narrow and large ring formation years. Horizontal dashed lines represent the average value. “+” and “−” represent the positive and the negative correlation between climate factors and tree-ring width, respectively.

**Figure 6:**
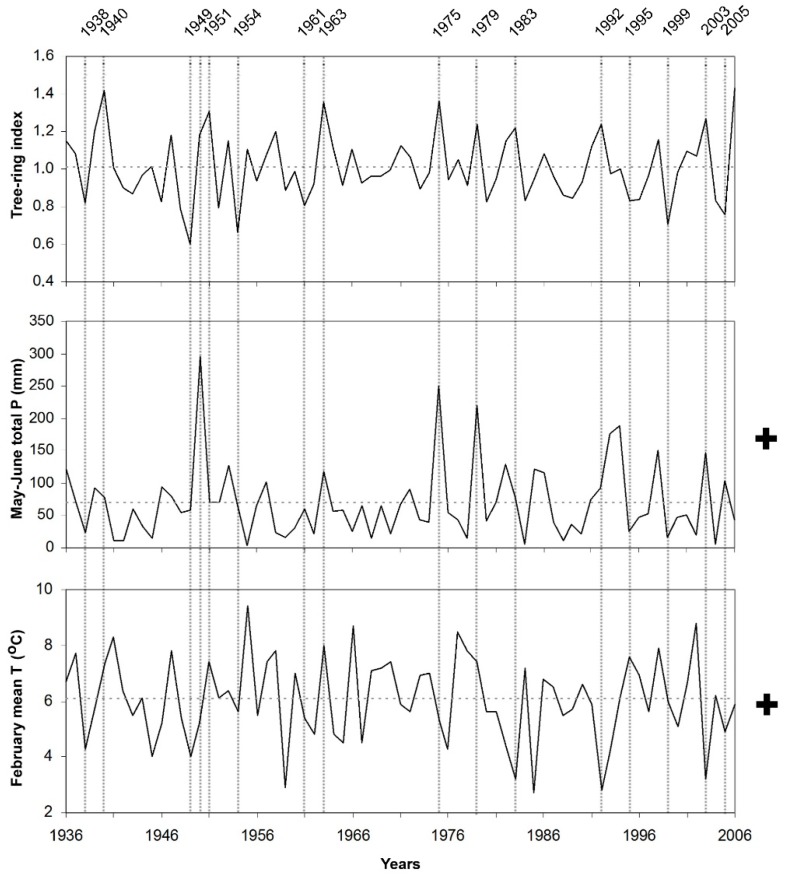
Residual chronology of lower limit of black pine on the north slope (CIA site), and limiting climate factors, which are May–June total precipitation and February mean temperature in the period of 1936–2006. Vertical dashed lines represent pointer years, which have very narrow and large ring formation years. Horizontal dashed lines represent the average value. “+” and “−” represent the positive and the negative correlation between climate factors and tree-ring width, respectively.

**Figure 7:**
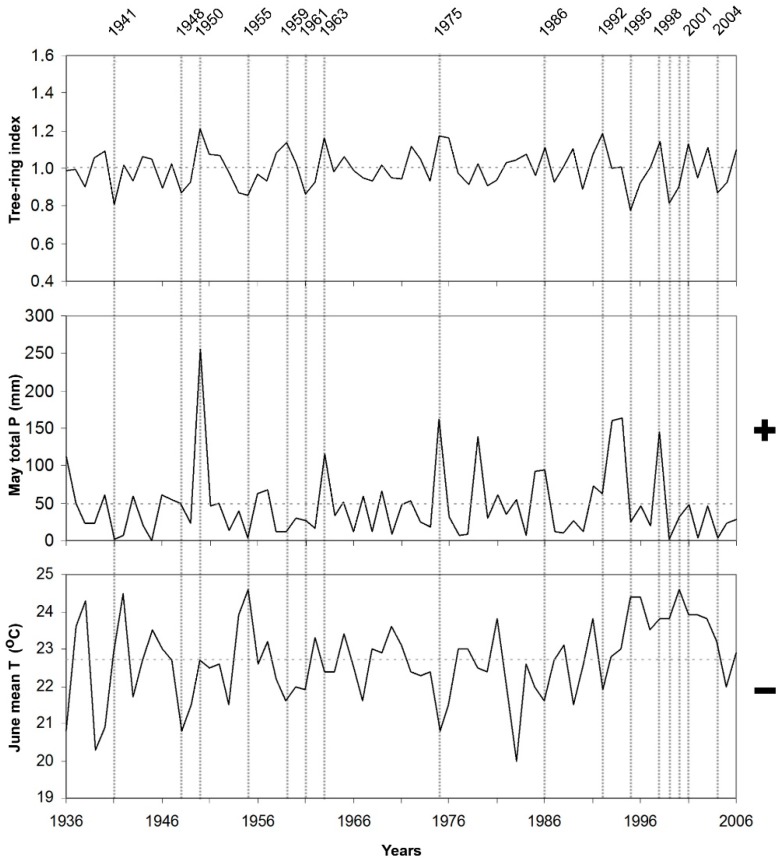
Residual chronology of upper limit of black pine on the north slope (CIU site), and limiting climate factors, which are May total precipitation and June mean temperature in the period of 1936–2006. Vertical dashed lines represent pointer years, which have very narrow and large ring formation years. Horizontal dashed lines represent the average value. “+” and “−” represent the positive and the negative correlation between climate factors and tree-ring width, respectively.

Response function results showed that temperature as well as precipitation, responsible for tree-ring width variations in the area. Accordingly, their effect should be taken into consideration together. We compare the most important limiting factors and tree-ring indices during the recorded period for each site separately, e.g., [24] (Figure 4, Figure 5, Figure 6 and Figure 7).

When temperature and precipitation data were compared with the tree-ring indices of black pine trees on Sandıras Mountain, it was seen that temperature and precipitation are both effective together in years when black pine annual rings are very narrow or very wide. For example, the largest ring width formed in 1975 on the lower limit on the south slope of the mountain (AGA chronology). In 1975, both lower mean temperatures and higher precipitation during May–June caused large ring formation. (Figure 4). The positive effect of the May–June period in 1975 was also clearly visible in the annual ring widths of the upper limit of black pine forests on the north slope of the mountain (CIU) (Figure 7).

On the other hand, depending on the elevation and slope in some years, only the negative or positive effect of precipitation/temperature affected the tree-ring growth. For example, the amount of high precipitation in the May–June of 1950 (especially for CIU) was the main driver that provides larger ring. (Figure 7).

In some years, the negative effect of temperature or precipitation in one period/month could be compensated by the positive effect of the other period/month, which means that the annual ring width could be close to the average ring width despite the negative effect. The examples of this were seen in the tree rings of 1955 and 1941 on the lower limit of black pine forest on the north slope of the Sandıras Mountain (CIA). In those years, May–June precipitation was well below the average. However, the fact that February temperatures were above the average, and accordingly the length of the vegetative period extended, provided annual ring width close to the average, even above the average (Figure 6).

## 4. Materials and Methods

### 4.1. Study Area

**Figure 8:**
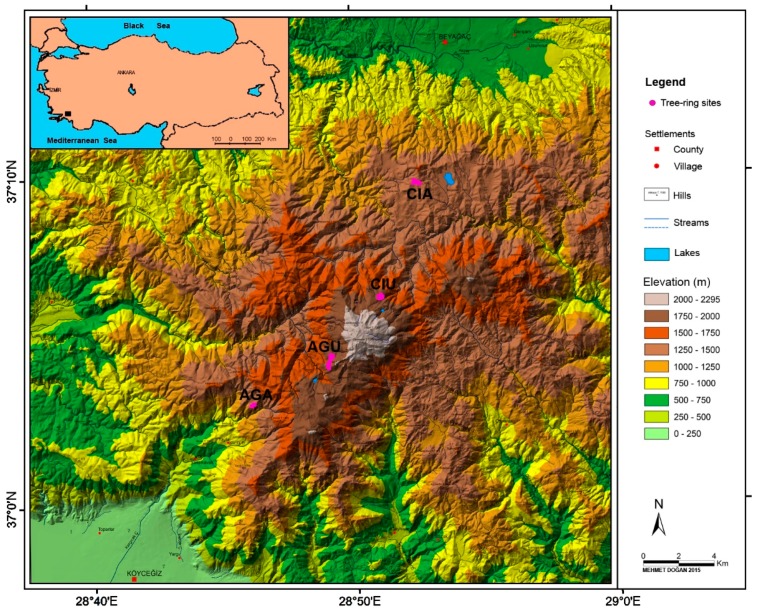
Location of sampling sites on Sandıras Mountain (magenta areas).

**Figure 9:**
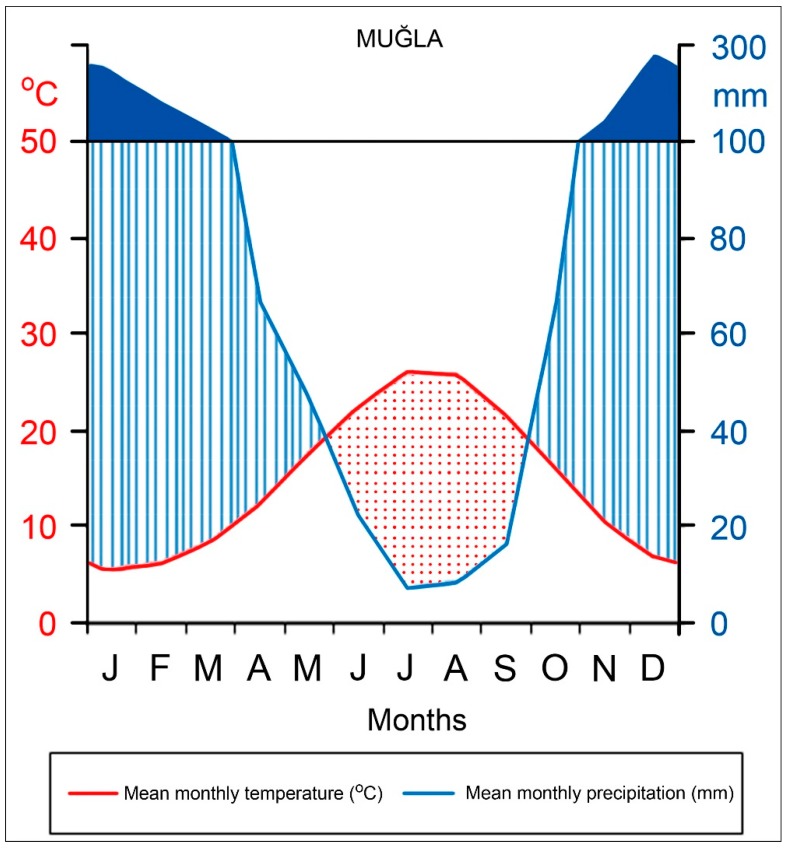
Monthly total precipitation and mean temperature values (1936–2006) (Climate diagram) of the Muğla meteorological station. Red and blue areas indicate dry and wet conditions respectively.

The study area is Sandıras Mountain located in southwest Turkey (Figure 8). Sandıras Mountain is one of the highest mountains of southwest Turkey, reaching up 2295 m (in Çiçekbaba hill). This mountainous area is one of the natural distribution areas of black pine and has one of the oldest black pine populations in Turkey. Monumental black pine stands and a large number of individual monumental trees can be observed between 1200 and 2000 m (especially north slope of the mountain). The Sandıras Mountain and its surroundings have a typical Mediterranean climate, with hot and dry summers and warm and rainy winters. Total annual precipitation and mean temperature (1936–2006) of Muğla meteorological station, which is the closest to the study area, were 1198.1 mm and 14.9 °C, respectively. The lowest precipitation is observed in July (7.1 mm) and August (8.5 mm), and the highest precipitation is observed in December (275.9 mm), while the highest mean temperature values occur in July (26.1 °C) and August (25.8 °C). During the year, the lowest temperature values are observed in January (5.4 °C) (Figure 9). The study area was located in higher elevation than Muğla meteorological station (646 m). Therefore, higher precipitation and lower temperature are expected in the study area.

### 4.2. Tree-Ring Chronologies and Climate Data

We used four black pine tree-ring chronologies built by Doğan and Köse [44] which represent upper and lower distribution limits in south and north slopes of Sandıras Mountain. Samples were taken from Eskere, Çiçekli (Denizli) for north slope of the mountain, and Köyceğiz, Ağla (Muğla) for south slope (Figure 8). Following steps were performed to build the chronologies by Doğan and Köse [44]: At least two increment cores per tree (from living trees) at breast height (1.30 m) were collected. Cores were glued onto grooved boards and sanded until annual rings were clearly visible. Cores were visually cross-dated with annual precision [i.e., each annual ring was assigned an exact calendar year of formation, using a combination of skeleton plotting [45] and the list method [46]. Tree-ring widths were measured with 0.01 mm precision using LINTAB-Tsap measurement system (RinnTech, Germany). The COFECHA software, which uses segmented time-series correlation techniques, was used to test the accuracy of measurements [47,48]. In total, we used 63 trees and 130 samples from four sites [for AGA site 15 trees and 31 samples, for AGU site 16 trees and 33 samples, for CIA site 16 trees and 32 samples, for CIU site 16 trees and 34 samples (Table 1)]. Each tree-ring measurement series were standardized by fitting a negative exponential regression equation. Then, loworder autoregressive models were applied to standardized series. Bi-weight robust mean was used to build a site chronology [49,50,51]. All these analyses were performed using ARSTAN software [49]. The mean sensitivity, which is a metric representing the year-to-year variation in ring width [2], was calculated for each chronology and compared (Table 1). Summary site information and statistics of the chronologies were given in Table 1 [44]. Monthly total precipitation and mean temperature records of Muğla Meteorological Station (1936–2006) were used in the analysis.

### 4.3. Identifying Relationship between Tree Growth and Climate

The relationships between tree-ring width and climate were identified using response function analysis [2]. Response function coefficients are estimates obtained by the multivariate technique of principal components that allows the use of correlated independent key events and tree growth indices as key responses [2,9]. The advantage of this method is that it removes the correlations between climate variables and converts them into principal components, which are orthogonal and uncorrelated [2,15]. Mean temperature and total precipitation values were arranged from previous October to current October (duration of the biological year). Response function coefficients were calculated for each site separately using DENDROCLIM2002 software [52]. We performed hierarchical binary clustering to classify the response functions into meaningful groups [2,8] using MATLAB software. Calculated response function coefficients of each site were used in the analysis. Similarity measures were calculated based on Euclidian distances.

## 5. Conclusions

In this study, we identified the most important climate factors affecting the radial growth of black pine distributed at the different elevations and slopes of Sandıras Mountain. Black pine trees in Sandıras Mountain responded positively to warmer temperature and high precipitation at the beginning of the growing season as it was in other areas in Western Anatolia. As high summer temperatures exacerbated drought, it affected radial growth negatively. Hierarchical cluster analysis made clear that elevation differences rather than aspect, was the main factor responsible for the formation of the clusters. Due to the mountainous terrain of the study area, the changing climatic conditions (air temperature and precipitation) affected the tree-ring width differently depending on elevation. The chronologies located on lower and upper forest limits of the mountain were separated in terms of their climate response. The trees that grow on the lower limit of the black pine forest were more sensitive to the variability in the climate than the trees that grow on the upper limit of black pine forest. However, the trees on the upper limit of black pine forest were more sensitive to winter precipitation at the southern slope (snowfall). High snow cover due to high snowfall caused the length of the vegetative period to shorten and the annual ring growth of trees to be limited.

## Figures and Tables

**Table 1:** Site information and summary statistics for site chronologies; from the ARSTAN program [44]. The lower elevation site chronologies (highlighted in bold) were more sensitive to climate variability than the higher elevation site chronologies.

	Site Code	Site Name	No. of the Trees/Cores	Aspect	Elevation	Time Span	Mean Sensitivity	Variance in First Eigenvector
South slope of Sandıras Mountain	AGA	Köyceğiz (Ağla) lower limit of black pine forest	15/31	S	1310–1370	1770–2010	0.22	47.37
	AGU	Köyceğiz (Ağla) upper limit of black pine forest	16/33	S and SW	1815–1890	1712–2010	0.16	33.98
North slope of Sandıras Mountain	CIA	Beyağaç (Eksere/Çiçekli) lower limit of black pine forest	16/32	S	1395–1425	1427–2010	0.27	56.15
	CIU	Beyağaç (Eksere/Çiçekli) upper limit of black pine forest	16/34	S	1805–1850	1191–2010	0.14	23.86
